# Measurements of the Specific Heats, *C_σ_*, and *C_v_* of Dense Gaseous and Liquid Ethane^*^

**DOI:** 10.6028/jres.080A.069

**Published:** 1976-10-01

**Authors:** Hans M. Roder

**Affiliations:** Institute for Basic Standards, National Bureau of Standards, Boulder, Colorado 80302

**Keywords:** Constant volume, ethane, heat capacity, liquid, saturated liquid, specific heat, vapor

## Abstract

The specific heats of saturated liquid ethane, *C_σ_*, have been measured at 106 temperatures in the temperature range 93 to 301 K. The specific heats at constant volume, *C_v_* have been measured at 19 densities ranging from 0.2 to 3.1 times the critical density, at temperatures between 91 and 330 K, with pressures to 33 MPa, at 200 PVT states in all. The uncertainty of most of the measurements is estimated to be less than 2.0 percent. As the critical point is approached the uncertainty rises to about 5.0 percent. The measurements were performed to provide input data for accurate calculations of the thermodynamic properties for ethane. They are believed to be the most comprehensive specific heat measurements available for the liquid and vapor states of ethane.

## 1. Introduction

For the calculation of the thermodynamic properties of a fluid, properties such as internal energy, enthalpy, entropy, and velocity of sound, especially at temperatures less than critical, one needs either the latent heat of vaporization or a specific heat along a path traversing the temperatures of interest. Heat capacity measurements are much easier to make than latent heat measurements, and they are not restricted to the liquid-vapor curve but can be made at temperatures and densities in the single phase fluid region as well.

For ethane, the specific heat of the saturated liquid, *C_σ_*, was measured from 93 to 301 K, and specific heats at constant volume, *C_v_*, were measured on 19 isochores with densities ranging from 1.5 mol/l to 21 mol/l with temperatures from 91 to 330 K and pressures up to 33 MPa [1].^1^

In a forthcoming publication Goodwin [2] uses the present results together with extensive *PVT* data to construct a complete thermodynamic network for ethane from the triple point to 600 K with pressures up to 70 MPa.

## 2. Experimental Method

The basics of the specific heat experiment are deceptively simple. The heat capacity of a sample of fluid is determined in principle as follows. A sample holder is filled with a known amount of sample, *N*, and is placed in an adiabatic environment. If we now apply a carefully measured amount of heat, *Q*, to the sample holder, then the temperature of sample and holder will rise to a new value, from an initial temperature, *T*_1_, to a final temperature, *T*_2_, the change in temperature being Δ*T*. To obtain the heat capacity of the sample we must account for the heat absorbed by the container. This is accomplished by conducting a second experiment with the sample holder empty to find the heat capacity of the empty container, *C*_0_. With

$$C_{0}=\frac{Q_{\text{MT}}}{\Delta T_{\text{MT}}}$$ (1)

the desired specific heat of the sample can be calculated from

$$C=\frac{Q-C_{0}\cdot \Delta T}{\Delta T\cdot N}.$$ (2)

Thus, the parameters we must measure in the experiment are *C*_0_, *Q*, Δ*T*, and *N.*

## 3. Apparatus and Procedures

**Figure 1:**
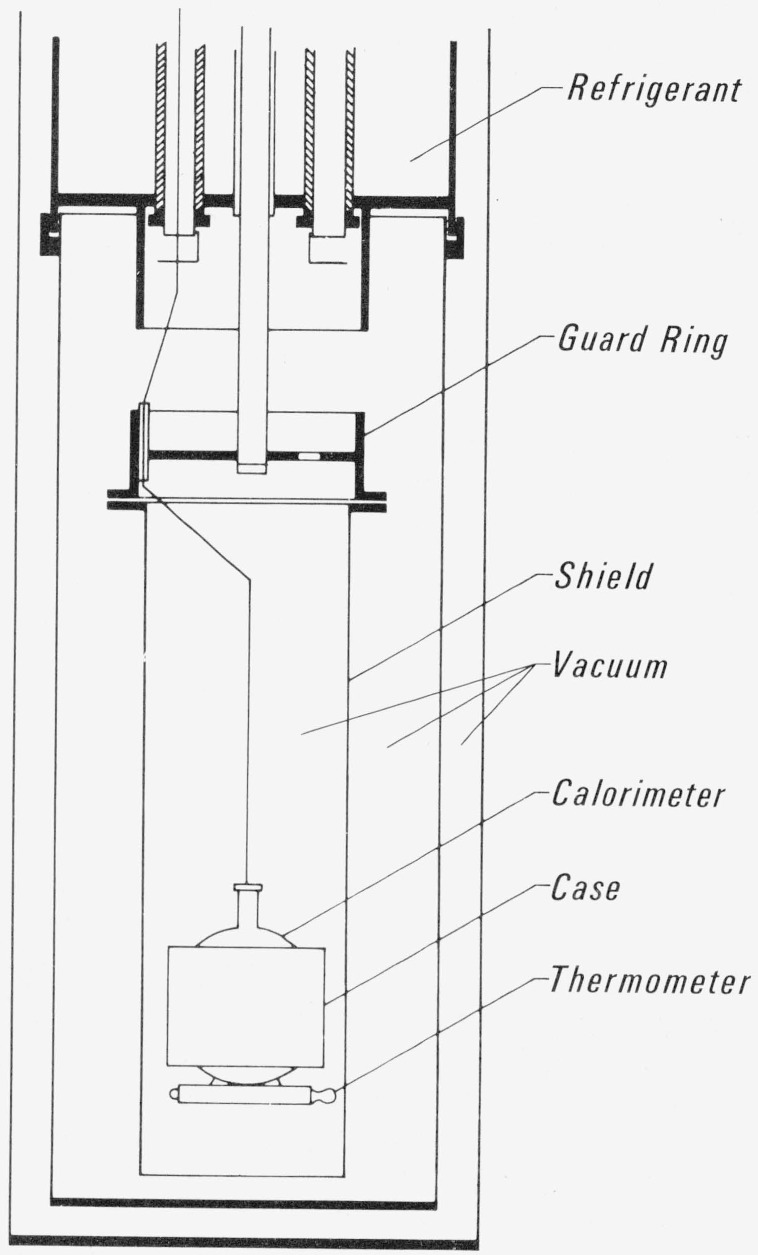
Calorimeter and cryostat.

The apparatus used is a constant volume adiabatic calorimeter fully described by Goodwin [3]. The essential features are a spherical sample holder, a filling capillary, a heating/cooling interceptor guard ring, an adiabatic shield, and a platinum resistance thermometer mounted on the sample holder. Calorimeter and cryostat are shown in figure 1. The refrigerants used were liquid nitrogen, ice, and cold water. The instrument has been used to measure the specific heats, *C_σ_* and *C_v_*, of hydrogen [4, 5], oxygen [6, 7], fluorine [8, 9], and methane [10]. Measurements of the heat of fusion and of the solid-solid transition in ethane with this apparatus have been reported elsewhere [11]. Minor modifications to the system have been described by Goodwin and Weber [6].

The major experimental parameters are *Q*, Δ*T*, and *N.* These are measured as follows. We obtain the calorimetric heating rate from nearly simultaneous readings of the potential and current applied to the calorimeter heater. The time of the heating interval is measured by an electronic counter triggered by the potential across the calorimeter heater. Temperatures are measured with the platinum resistance thermometer. The thermometer was calibrated by the NBS Temperature Section. Temperatures are on the IPTS–68 scale. The temperature of the adiabatic shield and guard ring are controlled to the sample temperature with difference thermocouples and automatic power regulation. The amount of sample is determined from an observed temperature *T* and pressure *P* in the single-phase domain, from the bomb volume at this *T*, *P*, and from the fluid density derived from an equation of state [2].

The ethane used in these experiments was commercially available research grade with minimum purity certified by the supplier at 99.98 percent. This purity was verified by chromatographic analysis.

The procedures used for measurement of the empty calorimeter, for loading of the sample, and for the specific heat measurements are the same as those used previously [4–10] except for filling the sample holder at low densities, and in the sequence of measurements. Differences in the filling of the calorimeter arise because the critical temperature of ethane is above room temperature. The ethane supply tank is normally at room temperature, about 296 K and the corresponding supply pressure, vapor pressure, is about 4 MPa. Fillings with liquid densities down to 12 mol/l are determined by selecting the temperature of the calorimeter, as before. However, different techniques had to be used to achieve densities below 12 mol/l. One was to raise the filling pressure by placing the ethane supply tank in a hot water bath, up to 40 °C. The other was to fill the sample holder around 12 mol/l, heat it to a temperature above critical, and then bleed it in small increments down to the desired density.

The sequence of measurements adopted was to conduct the *C_σ_* and *C_v_* measurements with a single filling rather than with different fillings as was the practice before. In this scheme the sample holder is filled to a known density in the single phase region and is then cooled to a temperature where both liquid and vapor are present in the calorimeter. Heating intervals are applied to the two phase sample, the data reduction yields values of *C_σ_.* During these measurements both liquid and vapor densities are changing, gradually filling the sample holder. From that point on the data reduction is carried out to yield values of *C_v_.* The heating interval in which the sample holder contains both two phase and single phase fluid is called the “breakthrough” point. A sharp change in rate of temperature rise can be seen on a recorder trace of calorimeter temperature, and both guard ring and shield heaters show a slight “bump” on the recorder traces of the differential thermocouples corresponding to a change in power requirement.

## 4. Calculations and Adjustments

The data reduction applicable to this experiment has been described in detail by Goodwin and Weber [6, 7]. However, for ethane the separate programs of *C_σ_* and *C_v_* were combined and a phasefinder developed that would pinpoint the “breakthrough” point of each filling. The phasefinder, the filling conditions, and the *PVT* conditions at which each point was measured are based on the equation of state by Goodwin [2].

One of the primary experimental parameters is the total amount of sample in the system, *N.* The pressure and temperature at filling are measured, the corresponding density is calculated from the *PVT* surface, and *N* is evaluated from a knowledge of the calorimeter volume, and the various ancillary volumes such as capillary, connector, and valve volumes. As mentioned before, the critical point of ethane, at 305.33 K, is above room temperature. A number of fillings and experimental measurements were made between 305 and 330 K. For these runs the portion of the sample in the capillary is not negligible, and has to be accounted for accurately. All of the “nuisance” volumes were revised, in particular the valve volume which, nominally at room temperature, was larger than previously estimated. To partially alleviate the problem the valve was thermostated at 40 °C, and a variable valve temperature was included in the data reduction routines. The amount in the capillary is determined by assuming a temperature distribution along the capillary. This distribution was changed to accommodate a variable temperature at the valve end.

Several other corrections made in the programs are reviewed briefly. The calorimeter volume depends both on temperature, thermal expansion, and on pressure [4, 6]. Since the sample holder is a thin stainless steel sphere it stretches as the pressure increases. Thus, in a *C_v_* measurement work is done by the sample due to the increase in sample volume. This correction developed by Walker [12] ranges from 0.5 to 5 percent of the resulting *C_v_* value. However, it can be made accurately. The density for each *C_v_* measurement is calculated from the filling density after correcting for sample holder expansion and the amount compressed into the filling capillary [7]. In the case of a *C_σ_* measurement the effects of the latent heat of vaporization and heat absorbed by the vapor must be subtracted [4, 6, 8]. This type of correction has been derived by Hoge [13].

It is worthwhile to mention that of the three state variables, pressure, temperature, and density, only temperature is measured during the measurement of a specific heat point. The amount of sample in the calorimeter is used to establish the density and pressure at the mean temperature of the experiment. While the total amount of sample remains constant, the distribution between calorimeter and capillary changes from point to point because the calorimeter volume changes with temperature and pressure. Thus, while the results for *C_v_* are corrected to be a true *C_v_* the measurements of a given run are made at slightly changing mean densities.

Curvature adjustments have been made for the *C_σ_* values at temperatures above 101.5 K. Adjustments to the experimental gross heat capacity-liquid and vapor-range from 0.002 J/mol-K to 0.366 J/mol-K, or up to 0.16 percent of the total value of *C_σ_.* Curvature adjustments for the values of *C_v_* were not significant, and were, therefore, omitted.

## 5. Heat Capacity of the Empty Calorimeter

Early estimates revealed that under the best of circumstances 50 percent of the applied heat is required for the calorimeter; for the very low densities at the highest temperatures up to 93 percent of the heating goes to raise the temperature of the calorimeter. Since the critical temperature of ethane is 305.33 K it appeared desirable to make at least some of the *C_v_* measurements at temperatures above critical. An upper limit of 338 K is imposed by the fact that the platinum resistance thermometer is mounted with Wood’s metal, which melts at 65 °C. Measurements on the other fluids had been carried out to only 300 K, therefore, an extension of the measurements on the empty calorimeter were indicated.

Remeasuring the heat capacity of the calorimeter *C*_0_ provided an opportunity to conduct additional checks of the system with regard to systematic errors, and to see if the precision of the measurements could be improved. The measurements of the heat capacity of the empty calorimeter included large and small Δ*T*’s from 8 to 0.5 K; large and small applied powers, from 1.0 to 0.23 W; runs with deliberate temperature offsets in both guard ring and shield temperatures, 3 K (100 *μ*V) hot and cold; as well as different coolants in the refrigerant tank, runs 2, 3, 4, with ice and runs 5, 6, 7 with liquid nitrogen. The results of these measurements, some 92 points, are shown in table 1. The applied temperature differences are small enough so that a curvature correction is not required. Intercomparison of the data is achieved by using the functional representation developed by Goodwin and Weber [6].

$${\mathrm{Log}}_{e}\left(C_{0}/50\right)=\sum_{i=1}^{8}C_{i}\cdot {\left(100/T\right)}^{i-1}.$$ (3)

Values of the coefficients, *C_i_*, are given in the heading of table 1.

Points 208 through 304 are included in table 1 to show the most extreme variation in Δ*T*. They were not used to obtain the coefficients, *C_i_* because during these runs one of the d.c. amplifiers had a large bias which was not corrected until the start of run 4. The analytical curve represents the heat capacities of the empty calorimeter with an imprecision of 0.07 percent. To the level of 0.1 percent in C_0_ there are no discernible systematic errors that can be related to the size of the Δ*T*, the size or rate of the applied heating, the temperature gradient of the capillary, or to temperature errors in shield or guard ring systems. The agreement of the present values with those measured by Goodwin and Weber [6] is well within the imprecision of the separate measurements. In the temperature range 87 to 320 K the uncertainty in the quantity (*Q−C*_0_Δ*T*)/Δ*T* will range from 0.04 to 0.08 J/K due to the uncertainties in *C*_o_ alone.

## 6. Results

**Figure 2:**
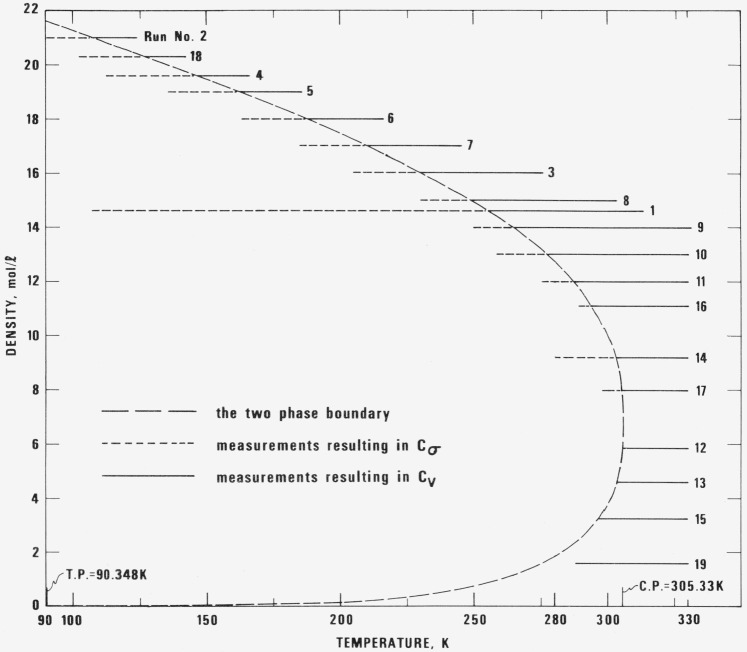
The locus of experimental runs in the density-temperature plane. — — — the two phase boundary - - - - - measurements resulting in *C_σ_* _________ measurements resulting in *C_v_*

The results to be presented include values for the specific heat of saturated liquid ethane, values for the specific heat of single phase ethane, both in compressed liquid and in gaseous states, and a limited set of measurements on methane, made for purposes of comparison. As mentioned above, both *C_σ_* and *C_v_* measurements were made during a single filling. Table 2 gives the loading conditions for all experimental runs; the runs are shown in density-temperature coordinates in figure 2. Temperature and pressure, obtained by computation from laboratory observations, are in effect direct measurements. The volume of the calorimeter is computed, the density is obtained from the equation of state [2]. The total number of moles, *N*, includes the amount in capillary and valve with the upper stem temperature equal to the indicated valve temperature. Breakthrough density and temperature define the point on the saturation boundary applicable to the run in question. The values are calculated from the equation of state, the loading conditions and the vapor pressure by considering the variation of calorimeter volume with temperature and pressure.

### 6.1. Performance Tests: The Specific Heats C_0_ and C*_v_* of Methane

Prior to making measurements on ethane we made a limited set of measurements on methane. The purpose was to check on the operation of the instrument by comparison to the values previously measured by Younglove [10]. Several values of CV and 29 values of *C_τ_* were measured at three different filling densities and at widely differing temperatures. Results and comparisons are shown in table 3. Two conclusions can be drawn from the measurements on methane. One is rather surprising, namely that the values of the specific heats calculated from the raw data will differ, if slightly different *PVT* surfaces are used in the data reduction process. The other is expected, namely that the values of the specific heats depend directly on the values measured for the heat capacity of the empty calorimeter.

To calculate the present results, which are shown in column 5 of table 3, we used Goodwin’s most recent formulation of the *PVT* surface of methane [14]. Since Younglove used a different, earlier formulation [15], a second calculation of our results using the earlier *PVT* surface is shown in column 6 of table 3. One of the most important differences between these two *PVT* surfaces is the assignment of critical density, 10.0 mol/l for reference [14], and 10.15 mol/l for reference [15]. The intercomparison of the two calculations is given in column 7. Clearly, both *C_σ_* and *C_v_* are sensitive to the *PVT* surface used in the data reduction process. Furthermore, the differences vary from point to point on the *PVT* surface. Recomputing all of Younglove’s results with the two different *PVT* surfaces leads to maximum differences of .08 percent in both *C_σ_* and *C_v_*. Accordingly, the most consistent way to compare the present results with those of Younglove [10] is to use the same *PVT* surface. This comparison involves columns 6 and 10 of table 3, with differences given in column 11. The disagreement between the two experiments runs from −1 to +1 percent. Several explanations were considered, only one of which is displayed in table 3. There exists a consistent offset, 0.4 percent, between the *C*_0_ measured in the course of this experiment and that measured by Younglove [10]. In column 8 we have calculated our present results on methane using Younglove’s [10] values for *C*_0_ and Goodwin’s earlier formulation of the *PVT* surface [15]. Column 9 shows the departure of the values in column 8 from those in column 6. Finally, a comparison of columns 9 and 11 suggests if not quantitatively, then at least qualitatively, that indeed the difference in the values of the *C*_0_’s is the explanation for the difference between the present measurements and those of Younglove [10].

### 6.2. The Specific Heats, *C_σ_* of Saturated Liquid Ethane

**Figure 3:**
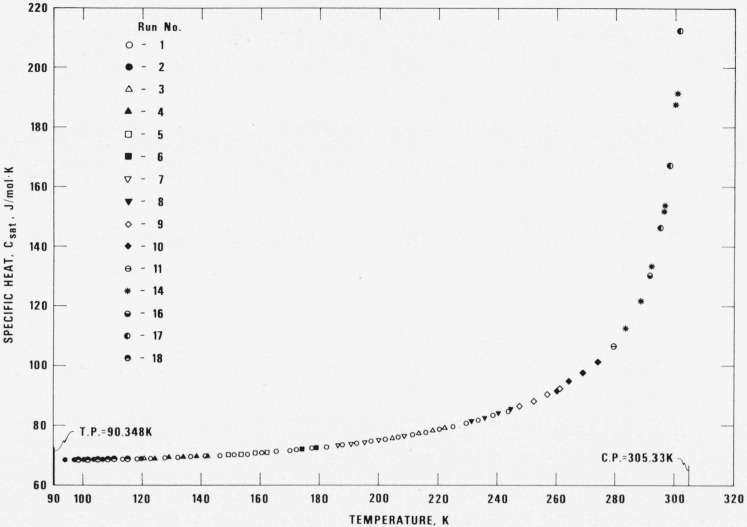
The specific heats, *C*_σ_, of saturated liquid ethane.

The specific heat of saturated liquid ethane was measured for 106 temperatures. The lowest was 93.7 K, the triple point is 90.348 K, the highest temperature was 301.5 K, the critical point is 305.33 K. Values of *C_σ_* along with the experimental conditions, experimental parameters, and the various correction terms are given in table 4. A plot of *C_σ_* is shown in figure 3. The measurements for *C_σ_* were made with Δ*T* between 3 and 5 kelvin, and with loadings such that at each temperature *C_σ_* is defined by at least two different fillings (see column 11, table 4). Curvature corrections were necessary only at temperatures above 101 K. The results for *C_σ_* are represented with an analytical equation as follows:

$$C_{\sigma }=C_{1}+C_{2}T+C_{3}T/{\left(T_{c}-T\right)}^{0.6}+C_{4}/T+C_{5}/T^{2}$$ (4)

where *T_c_* is the critical temperature, 305.33 K and values of the coefficients are given in the heading of table 4. Values calculated from eq. (4) and differences between experimental and calculated values expressed in percent are also given in table 4. The standard deviation of the entire fit is 0.3 J/mol-K. For temperatures below 260 K the imprecision in the experiment is ±0.1 percent, not much larger than that experienced for measurements of the empty calorimeter. Considering all sources the estimated uncertainty in the measured value of *C_σ_* is about 0.5 percent generally, increasing to about 5 percent within a few kelvin of the critical point.

Comparison with the earlier measurements of Wiebe et al. [16], and Witt and Kemp [17] is made using eq (4) for interpolation. Differences in *C_σ_* are shown in table 5. They are negligible at low temperatures but increase gradually to 5 percent at the highest temperatures of comparison. The explanation of the differences lies in the different PVT surfaces used to evaluate the experimental data and correction terms, in particular the rather large difference in assignment of the critical density, 6.80 mol/l this experiment and 6.99 mol/l for the other authors.

### 6.3. The Specific Heats, C*_v_*, of Dense Gaseous and Liquid Ethane

**Figure 4:**
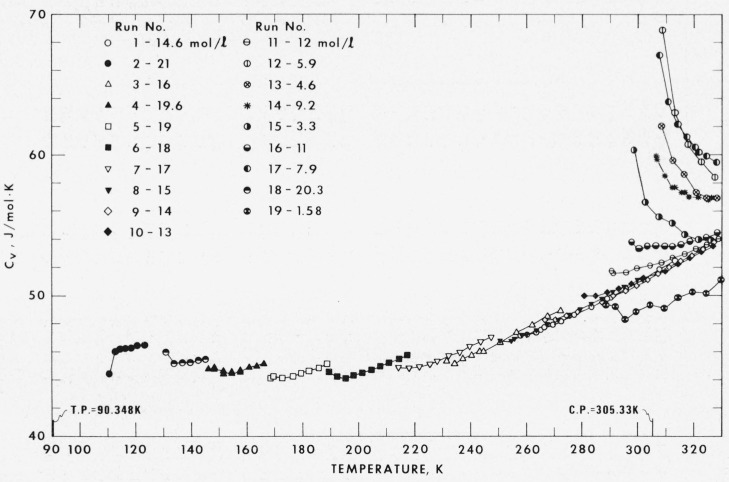
The specific heats, *C*_v_, of dense gaseous and liquid ethane.

The specific heats at constant volume were measured at 19 densities ranging from 0.2 to 3.1 times the critical density, at temperatures between 91 and 330 K, and with pressures to 33 MPa. As shown in figure 2, a given density is limited either by the maximum allowable system pressure, about 35 MPa, which in turn leads to a maximum pressure of 33 MPa at the mean temperature of the experiment, or by the upper limit in temperature, 330 K. Values of *C_v_* along with the experimental conditions, the major experimental parameters, and the correction term are given in table 6. The temperature and density dependence of *C_v_* is illustrated in figure 4. For a wide range of densities to either side of the critical density the specific heat increases sharply as the coexistence enevelope is approached. At liquid densities far removed from critical the temperature dependence is relatively weak.

Representation of the specific heats has been achieved by Goodwin [2] who correlates the available *PVT* data, the specific heats of the ideal gas, the specific heat of the saturated liquid from this experiment, and selected values of *C_v_* from runs 1, 8, and 9. Values of *C_v_* calculated from his equation of state and differences between experimental and calculated values expressed in percent are given in table 6. A scan of column 7 in table 6 reveals that the agreement between experimental and calculated values is excellent over much of the *PVT* surface, i.e., a nominal 2 percent or less. It is only in the region near the critical point where the experimental heat capacities increase drastically that the representation is not able to match the experimental surface of *C_v_*, departures reach values of up to 18 percent. The agreement for the very lowest density, run 19, is particularly gratifying since for this run the experimental uncertainty in *C_v_* is about 2 percent, whereas the calculation οf *C_v_* from ideal gas and a very small *PVT* contribution should have very little error attached to it.

Experimental measurements of specific heats have been made by other authors [18,19], however these measurements are measurements of the specific heat at constant pressure, *C_p_.* The values can be compared only indirectly to the present measurements through the use of a *PVT* surface. Since *C_p_* measurements are normally made along isobars while *C_v_* measurements are made along isochores, the most appropriate *PVT* states for comparisons are at those values of pressure and density common to both sets of data. For the results of Furtado [18] and the present measurements this intercomparison is given in table 7. The pressures are taken from reference [18], the densities from the present measurements. A temperature corresponding to the *P* and *ρ* of an intersection is obtained from the equation of state [Goodwin, 2], and experimental values of *C_v_* and *C_p_* are interpolated from the two sets of data. The comparison is completed by calculating a value of *C_p_* from

$$C_{p}=C_{v}+\frac{T{\left(\frac{\partial P}{\partial T}\right)}_{\rho }^{2}}{\rho^{2}{\left(\frac{\partial P}{\partial \rho }\right)}_{T}}.$$ (5)

The second term in equation 5 is the contribution from the *PVT* surface. It is clear from table 7 that in almost all cases the *PVT* contribution to *C_p_*-calculated is as large or larger than the value of *C_v._* The mean deviation between calculated and experimental *C_p_* for the 50 intersections is just under 2 percent. This implies that the thermodynamic consistency between experimental *C_v_* and *C_p_* measurements is indeed excellent, i.e., at least as good as 2 percent, but quite probably much better than that for two reasons. First, a large part of the total discrepancy must be assigned to errors in the *PVT* surface derivatives. Second, some of the values presented by Furtado [18] may be in error by as much as 10 percent. A detailed example is as follows.

In our table 7 a comparison is made at 6.8948 MPa (1000 psia) and 298.929 K (78.40°F). Furtado’s closest smoothed value of *C_p_* taken from his table VIII-5 at 6.8948 MPa (1000 psia) and 299.817 K (80°F) is 111.84 J/mol-K (0.889 BTU/lb-°F). This value changes to 124.06 J/mol-K if interpolated from his table VIII-3, a table of smoothed enthalpies, or to 122.4 J/mol-K if interpolated from his figure VIII-6, a plot of *C_p_* versus temperature for the 1000 psia isobar. Thus the inconsistencies in Furtado’s values, depending on how they are obtained, are at times as large at 10 percent.

## 7. Discussion

It is readily apparent that accurate values of *C*_0_ are essential if we wish to obtain accurate values of either *C_σ_* or *C_v_.* A change of 0.1 percent in *C*_0_, for example, will result in a change of 1 percent in the values of *C_v_* calculated for run 19. The temperature increment, Δ*T*, is evaluated at the middle of the heating interval by extrapolating the temperature drift rates just before heating and after an equilibrating time has elapsed. Since the drift is linear the statistics of the extrapolation can be used to estimate an uncertainty in the Δ*T.* For the first 14 points of *C*_0_ the average slope uncertainty was 0.19×10^−3^ K/min, since the average elapsed time to the center of the measurement interval is about 20 min, the average uncertainty in Δ*T* turns out to be ±0.004 K. This in turn implies that if we seek 0.1 percent precision in the specific heats the Δ*T* must be 4 K or larger. The choice of Δ*T* as shown in tables 4 and 6, was based on this consideration and on the idea that there ought to be at least 5 points per experimental run. The imprecision in the temperature time data is attributed to the exact setting or resetting of the platinum thermometer current rather than potentiometer inaccuracy. Potentiometer inaccuracy was actually reduced from values given by Goodwin and Weber [6] to a maximum of 0.003 K by consideration of a potentiometer calibration. Heat leak to and from the sample is estimated to be less than 0.1 percent by considering the difference in drift rates before heating and after equilibration has been reached. Shield temperatures lag at the start of the heating interval by about 0.02 K. They lag again at the end of the heating interval after the power is turned off. The two lags compensate to produce a nearly adiabatic environment during the entire heating interval. Deliberate changes of temperature along the capillary were introduced to see if the applied heat, and therefore the results could be changed. In run 14 points 1401–1413 were obtained with liquid nitrogen in the refrigerant tank. These points were duplicated for 1414–1425 using cold water as coolant. The results, as shown in tables 4 and 6, are virtually identical. However, when we applied deliberate heating to the capillary, actually quite drastic heating 100 ma to a 140 Ω heater, the results changed. Points 508–517 differ from those obtained in a duplicate run 520–527 without heating the capillary by 1 percent at the lowest temperature. The difference disappears entirely at the highest temperature of the run. However, rather than changing the applied heat, heating the capillary apparently changes the distribution of sample between calorimeter and stem.

The same problem, distribution of sample between calorimeter and stem, is thought to give rise to the curvature of the runs. Runs 18, 4, 5, 6, and 7 show a definite curvature as to two phase boundary is approached, see figure 4, if compared to the values calculated by Goodwin [2]. The curvature seems to abate at pressures above the critical pressure, or at a point where mass change between calorimeter and stem has stabilized. It is possible that a heat of vaporization correction to *Q* should be included for the *C_v_* calculation as long as sample is being transferred from calorimeter to stem. It should be noted that the correction term, column 13 of table 6, is irregular for the first few points of the runs in question.

Sample distribution is also thought to explain the departure of point 208 from the rest of run 2. The possibiity exists that for run 208 the capillary was frozen, because if point 208 is recalculated with zero stem volume the value of *C_v_* is increased by about 2.5 percent.

We had hoped to employ the breakthrough points to resolve the problem of sample distribution. Experimental breakthrough temperatures agree well with calculated values; densities and total sample agree to a point where we are confident that the calorimeter volume has not changed. However, to calculate *C_σ_* and *C_v_* values from a breakthrough point requires that we know the time of breakthrough exactly in order to proportion the applied heat *Q.* There is simply too much lag in the response of the recorders to permit an accurate determination of the breakthrough time.

The imprecision in the experiment depends primarily on the imprecision of *C*_0_ and on the amount of sample since in most cases the Δ*T* is around 4 K. For liquid densities the imprecision from point to point along an isochore is about 0.1 percent with occasional differences as large as 0.3 percent. For densities less than critical the imprecision, the variation of *C_v_* point to point from a smooth curve, increases to about 1 percent. The inaccuracy or uncertainty of the present measurements is estimated from the comparison to the experiments of others and from the comparison to values calculated from the *PVT* correlation. We consider the excellent agreement between the present results and the experiments of others [10, 16, 17, 18], in particular the agreement with experimental values of *C_p_*, and we consider the results of deliberately introducing changes in the present experiment. It is difficult to see how systematic errors larger than about 2 percent for liquid densities or larger than 5 percent for vapor densities close to critical could remain undetected in the present experiment.

## Figures and Tables

**Table 1:** Heat capacity of the empty calorimeter Coefficients, eq [3]: C1 = 1.352617652, C2= −7. 469809247, C3 = 30.953781888, C4 = −76.348710878 C5 = 110.748213166, C6 = −94.987724059, C7 = 44.485629941, C8 = −8.756298569

Run No. — Point	Temperature	Power	Time	Heat, *Q*	Δ*T*	C_0_	C_0_ Calc.	Dev.	Remarks
								
No.	*K*	W	s	*J*	*K*	*J/K*	*J/K*	0/0	
									
208	300.294	0.23661	723.20	171.11	2.066	82.810	83.088	−0.34	Not used in fit
209	302.419	.23724	721.35	171.13	2.061	83.040	83.230	−.23	Not used in fit
210	303.809	.23720	185.20	43.93	.528	83.142	83.322	−.22	Not used in fit
302	275.050	.24587	181.92	44.73	.549	81.523	81.311	.26	Not used in fit
303	275.881	.24655	360.18	88.80	1.091	81.389	81.373	.02	Not used in fit
304	278.500	.93146	361.54	336.76	4.129	81.559	81.566	−.01	Not used in fit
401	279.032	1.00446	360.92	362.53	4.446	81.543	81.605	−.08	
402	283.489	1.00375	360.25	361.60	4.416	81.892	81.927	−.04	
403	287.955	1, 00340	361.62	362.85	4.414	82.213	82.243	−.04	
404	292.433	1.00341	360.78	362.01	4.387	82.526	82.555	−.04	
405	296.923	1.00302	361.68	362.77	4.385	82.733	82.862	−.15	
406	295.532	1.00384	362.13	363.52	4.397	82.683	82.767	−.10	
407	300.004	1.00318	360.49	361.64	4.357	83.010	83.069	−.07	
408	304.486	1.00292	361.54	362.60	4.357	83.232	83.367	−.16	
409	309.774	1.00062	361.05	361.27	4.320	83.636	83.712	−.09	Guard ring 3 K cold
410	314.120	1.00050	360.63	360.81	4.301	83.884	83.992	−.13	Guard ring 3 K cold
411	318.482	1.00075	360.05	360.32	4.278	84.235	84.269	−.04	Guard ring 3 K cold
412	309.649	1.00114	358.38	358.79	4.291	83.620	83.704	−.10	Guard ring 3 K hot
413	314.300	1.00111	360.84	361.24	4.298	84.044	84.004	.05	Guard ring 3 K hot
414	319.013	1.00083	361.65	361.95	4.286	84.442	84.303	.16	Guard ring 3 K hot
501	273.211	1.01274	363.70	368.33	4.535	81.219	81.173	.06	
502	277.724	1.01203	360.42	364.76	4.472	81.572	81.509	.08	
503	282.215	1.01194	361.01	365.31	4.462	81.871	81.835	.04	
504	286.704	1.01106	361.10	365.09	4.442	82.199	82.155	.05	
505	291.192	1.01099	361.06	365.02	4.423	82.520	82.469	.06	
506	295.681	1.01067	361.04	364.89	4.406	82.814	82.777	.04	
507	300.167	1.01023	360.65	364.34	4.383	83.132	83.080	.06	
508	304.708	1.00964	361.07	364.55	4.369	83.447	83.381	.08	
509	309.206	1.00927	360.48	363.82	4.346	83.711	83.675	.04	
510	314.458	1.00864	483.74	487.91	5.802	84.100	84.014	.10	
511	319.745	1.00766	360.77	363.53	4.306	84.418	84.349	.08	
601	127.080	.57391	482.10	276.68	4.659	59.390	59.270	.20	
602	131.608	.57368	482.80	276.97	4.566	60.662	60.644	.03	
603	136.098	.57391	482.50	276.91	4.470	61.945	61.920	.04	
604	142.318	.57390	899.00	515.94	8.114	63.588	63.562	.04	
605	150.056	1.00467	485.05	487.31	7.443	65.474	65.422	.08	
606	131.622	.58565	174.94	102.45	1.690	60.607	60.648	−.07	
607	134.090	.58574	347.99	203.83	3.322	61.354	61.360	−.01	
608	137.347	.58516	347.34	203.25	3.266	62.225	62.261	−.06	
609	139.657	.58460	174.57	102.05	1.623	62.897	62.877	.03	
610	142.810	.58371	518.78	302.82	4.755	63.686	63.686	.00	
611	147.463	.58495	514.99	301.24	4.647	64.827	64.819	.01	
612	152.806	.58497	693.56	405.71	6.141	66.064	66.039	.04	
613	161.128	.57559	605.25	348.38	5.143	67.734	67.782	−.07	
614	167.386	.57535	897.63	516.45	7.490	68.948	68.983	−.05	
615	174.725	.57518	899.07	517.13	7.355	70.309	70.284	.04	
616	175.502	1.01504	263.20	267.16	3.793	70.431	70.415	.02	
617	179.197	1.01430	262.02	265.77	3.742	71.022	71.025	−.00	
618	182.608	1.01317	261.33	264.77	3.703	71.509	71.567	−.08	
619	186.241	1.01301	262.22	265.63	3.686	72.065	72.122	−.08	
620	188.966	1.01269	136.12	137.84	1.899	72.580	72.525	.08	
621	190.803	1.01218	135.19	136.84	1.878	72.861	72.790	.10	
622	194.400	1.01244	392.67	397.55	5.425	73.275	73.295	−.03	
623	199.723	1.01223	392.50	397.30	5.372	73.962	74.009	−.06	
624	202.396	1.01658	516.29	524.84	7.062	74.323	74.353	−.04	
625	206.831	.28352	516.44	146.42	1.954	74.940	74.906	.04	
626	208.722	.28345	519.22	147.17	1.960	75.102	75.134	−.04	
627	212.843	.97721	517.07	505.29	6.685	75.583	75.619	−.05	
628	220.458	1.01519	654.30	664.24	8.686	76.476	76.469	.01	
629	226.417	.28269	920.69	260.27	3.377	77.059	77.096	−.05	
630	229.716	.28266	922.57	260.77	3.365	77.501	77.430	.09	
631	235.624	1.01464	658.73	668.38	8.567	78.014	78.006	.01	
632	232.939	.58665	783.62	459.71	5.909	77.793	77.748	.06	
633	238.781	.58629	784.29	459.82	5.872	78.313	78.304	.01	
634	244.575	.58619	780.71	457.64	5.807	78.810	78.833	−.03	
635	250.293	.58579	786.49	460.72	5.808	79.320	79.334	−.02	
636	256.055	.58558	784.73	459.52	5.756	79.836	79.821	.02	
637	261.790	.58544	785.31	459.75	5.723	80.339	80.287	.06	
638	267.500	.58482	782.99	457.91	5.665	80.832	80.737	.12	
639	273.202	.58484	785.46	459.37	5.657	81.207	81.172	.04	
640	277.804	.58479	786.09	459.70	5.639	81.520	81.515	.01	
641	283.507	.58451	784.38	458.47	5.594	81.956	81.928	.03	
642	289.440	.58427	785.21	458.77	5.567	82.403	82.347	.07	
643	295.144	.58405	784.02	457.90	5.529	82.822	82.741	.10	
644	87.956	.58825	329.66	193.92	4.522	42.888	42.881	.01	
645	92.450	.58814	346.38	203.72	4.502	45.250	45.272	−.05	
646	96.923	.58758	361.11	212.18	4.469	47.476	47.464	.02	
647	101.371	.58799	375.32	220.68	4.456	49.524	49.519	.01	
648	105.801	.58779	388.99	228.64	4.442	51.476	51.454	.04	
649	110.252	.58769	407.99	239.77	4.499	53.297	53.290	.01	
650	114.693	.58742	421.62	247.67	4.503	54.999	55.013	−.02	
651	119.179	.58700	435.77	255.80	4.519	56.604	56.645	−.07	
652	123.684	.58733	450.21	264.42	4.549	58.134	58.179	−.08	
653	128.143	.58676	450.00	264.04	4.435	59.538	59.601	−.10	
701	276.822	.58535	781.32	457.34	5.616	81.429	81.442	−.02	Guard ring 3 K cold
702	282.678	.58521	785.71	459.80	5.617	81.865	81.869	−.00	Guard ring 3 K cold
703	276.345	.58488	784.87	459.05	5.642	81.369	81.407	−.05	Guard ring 3 K hot
704	281.862	.58439	784.15	458.25	5.606	81.749	81.810	−.07	Guard ring 3 K hot
705	276.552	.58419	781.97	456.82	5.612	81.400	81.422	−.03	Shield 3 K cold
706	282.382	.58464	783.58	458.11	5.598	81.834	81.847	−.02	Shield 3 K cold
707	275.701	.58248	782.35	455.70	5.604	81.320	81.359	−.05	Shield 3 K hot
708	281.212	.58417	784.63	458.36	5.610	81.706	81.763	−.07	Shield 3 K hot

**Table 2:** Calorimeter loading conditions for the measurement runs

Run No.	Pressure	Density	Temperature	Calorimeter volume	Total sample	Valve temperature	Breakthrough conditions	
							Density	Temperature
								
	MPa	mol/l	*K*	cm^3^	mol	*K*	mol/l	*K*
								
1	4.0982	14.641	258.899	73.238	1.0730	296.45	14.659	254.048
2	3.8329	20.998	110.512	72.794	1.5289	296.45	21.013	108.627
3	4.0753	16.135	231.874	73.150	1.1811	296.45	16.157	227.207
4	3.8996	19.648	147.427	72.892	1.4326	296.45	19.664	144.870
5	2.3023	18.923	165.524	72.928	1.3802	314.65	18.932	163.802
6	3.8980	17.985	190.218	73.018	1.3135	314.55	18.001	186.776
7	3.4113	16.912	214.723	73.089	1.2363	314.27	16.925	211.275
8	4.0396	15.059	251.910	73.215	1.1028	314.19	15.071	247.239
9	3.9223	14.001	268.047	73.267	1.0261	314.16	14.010	263.866
10	3.9178	12.950	280.749	73.308	.9495	314.00	12.956	277.448
11	3.9875	11.978	289.559	73.339	.8786	313.42	11.981	287.411
12	5.3477	5.886	310.707	73.425	.4325	314.15	5.889	305.158
13	5.0159	4.610	308.612	73.414	3387	313.15	4.612	303.177
14	4.9503	9.179	304.928	73.401	.6740	313.59	9.181	303.179
15	5.4586	3.269	329.810	73.491	.2406	313.25	3.277	296.609
16	5.2186	11.045	299.922	73.387	.8109	313.25	11.053	294.671
17	6.0158	7.884	313.979	73.444	.5796	313.17	7.894	305.106
18	4.0370	20.298	129.892	72.845	1.4790	296.45	20.314	127.574
19	3.2471	1.583	319.878	73.431	.1163	313.78	1.587	274.258

**Table 3:** Results and intercomparison of the specific heats of methane

Run No. — point No.	Saturation conditions based on reference [14]			The specific heat, *C_σ_*, of saturated liquid methane						
				Results this paper calculated with *C*_0_ and *PVT* sources below					Interpolated from ref. [10]	
				*C*_0_: This paper *PVT*: Ref. [14]	This paper ref. [15]		Ref. [10] Ref. [15]			
				
	Vapor pressure MPa	Liquid density mol/l	Temperature *K*	J/mol-*K*	J/mol-*K*	Dev. col 5 to 6 0/0	J/mol–*K*	Dev. col 6 to 8 0/0	J/mol–*K*	Dev. col 6 to 10 0/0
										
118	0.1436	25.939	116.044	56.58	56.74	−0.27	56.97	−0.42	56.52	0.39
119	.1721	25.710	118.479	56.76	56.91	−.27	57.16	−.43	56.75	.29
120	.2219	25.365	122.089	57.29	57.44	−.27	57.70	−.44	57.19	.45

Run No. —point No.	*PVT* conditions based on reference [14]			The specific heat, *C_v_* of singlephase fluid methane						
				Results this paper calculated with *C*_0_ and *PVT* sources below					Interpolated from ref. [10]	
				*C*_0_: This paper *PVT*: Ref. [14]	This paper ref. [15]		Ref. [10] Ref. [15]			
				
	Pressure MPa	Density mol/l	Temperature *K*	J/mol-*K*	J/mol-*K*	Dev. col 5 to 6 0/0	J/mol-*K*	Dev. col 6 to 8 0/0	J/mol-*K*	Dev. col 6 to 10 0/0
										
101	6.4874	11.802	200.945	34.01	34.21	−0.60	34.62	−1.20	34.42	−0.61
102	6.7965	11.801	202.608	33.47	33.67	−.60	34.08	−1.21	33.88	−.63
103	7.2646	11.798	205.115	32.77	32.96	−.60	33.37	−1.23	33.04	−.23
104	7.8939	11.795	208.469	32.16	32.36	−.60	32.75	−1.22	32.45	−.29
105	8.6908	11.791	212.697	31.56	31.75	−.60	32.13	−1.20	31.69	.18
106	9.6540	11.786	217.783	31.12	31.31	−.60	31.67	−1.16	31.00	.98
107	4.6707	11.811	190.959	51.72	52.03	−.59	52.45	−.81	- - - - - - - - - -	- - - - - - - - - -
108	4.9409	11.809	192.489	40.10	40.34	−.60	40.76	−1.05	- - - - - - - - - -	- - - - - - - - - -
109	5.2290	11.808	194.092	37.79	38.02	−.60	38.44	−1.11	- - - - - - - - - -	- - - - - - - - - -
110	5.5236	11.807	195.713	36.47	36.69	−.60	37.11	−1.15	37.09	−1.09
111	5.8105	11.805	197.280	35.39	35.60	−.60	36.02	−1.18	35.79	−.54
112	6.1116	11.804	198.915	34.65	34.85	−.60	35.27	−1.19	35.24	−1.11
113	6.4166	11.802	200.564	34.09	34.30	−.60	34.71	−1.20	34.59	−.86
114	6.7237	11.801	202.217	33.41	33.61	−.60	34.03	−1.22	33.92	−.92
115	7.1915	11.798	204.724	32.87	33.06	−.60	33.47	−1.22	33.10	−.11
116	7.8235	11.795	208.095	32.24	32.43	−.60	32.83	−1.22	32.49	−.18
117	8.6216	11.791	212.330	31.63	31.82	−.60	32.20	−1.20	31.75	.21
201	6.5423	20.504	170.403	30.30	30.21	.27	30.44	−.74	30.63	−1.38
202	7.6896	20.499	172.193	30.33	30.26	.22	30.49	−.75	30.57	−1.03
203	9.4196	20.489	174.898	30.22	30.17	.16	30.40	−.77	30.51	−1.12
204	11.7168	20.479	178.506	30.16	30.11	.17	20.35	−.79	30.42	−1.01
205	13.9877	20.470	182.089	30.09	30.04	.17	20.28	−.80	30.33	−.98
301	6.8455	23.445	146.608	31.16	31.15	.04	31.20	−.48	31.50	−1.13
302	8.5366	23.438	148.358	31.13	31.12	.04	31.28	−.49	31.46	−1.07
303	10.1996	23.431	150.090	31.14	31.13	.04	31.29	−.50	31.42	−.92
304	12.6822	23.417	152.695	31.10	31.08	.05	31.25	−.52	31.35	−.85
305	15.9591	23.403	156.170	31.05	31.03	.07	31.20	−.54	31.27	−.76
306	19.5801	23.385	160.058	31.01	31.01	.03	31.18	−.57	31.18	−.56
307	23.5208	23.368	164.346	30.93	30.92	.05	31.11	−.60	31.11	−.60

**Table 4:** The specific heat, C_σ_, of saturated liquid ethane Coefficients, eq[4]: C1 = 0.2264822683E+02, C2 = 0.8796160711E−01, C3 = 0.1090640627E+01, C4 = 0.5371201054E+04, C5 = −0.2123389940E+06

Run No. — point No.	Saturation conditions			C_σ_ J/mol-K	C_σ_ Calc. J/mol-K	Dev. 0/0	Heat, *Q J*	Δ*T K*	C_0_ *J/K*	Sample mol	Calorimeter Volume cm^3^	Expansion + capillary correction J/mol-K	Vaporization+vapor correction J/mol-K	Curvature correction J/mol-K
	Vapor pressure MPa	Liquid density mol/l	Temperature *K*											
														
202	0.0000	21.558	93.712	68.27	68.14	0.18	504.66	3.358	45.910	1.5289	72.726	0.000	−0.000	
203	.0000	21.436	97.045	68.30	68.29	.02	504.23	3.318	47.520	1.5289	72.733	.000	−.000	
1801	.0000	21.398	98.095	68.44	68.32	.17	508.65	3.408	48.020	1.4790	72.735	.000	−.000	
204	.0000	21.317	100.322	68.45	68.40	.08	499.81	3.252	49.040	1.5289	72.740	.000	−.000	
1802	.0000	21.274	101.482	68.44	68.44	.01	507.43	3.365	49.570	1.4790	72.742	.000	−.000	
205	.0000	21.198	103.565	68.46	68.50	−.06	503.65	3.246	50.490	1.5289	72.747	.000	.000	
1803	.0000	21.152	104.828	68.53	68.54	−.01	508.13	3.334	51.040	1.4790	72.750	.000	−.000	
206	.0000	21.080	106.788	68.35	68.59	−.34	502.83	3.215	51.870	1.5289	72.754	.000	.000	
1804	.0001	21.031	108.142	68.60	68.62	−.04	507.45	3.298	52.430	1.4790	72.757	.000	−.000	
127	.0001	21.026	108.287	68.60	68.63	−.04	622.67	4.938	52.490	1.0730	72.757	.000	−.000	
1805	.0001	20.943	110.531	68.66	68.68	−.04	698.13	4.506	53.400	1.4790	72.762	.000	−.000	
401	.0001	20.931	110.862	68.72	68.59	.04	751.16	4.943	53.530	1.4326	72.763	.000	−.000	
128	.0001	20.846	113.180	68.73	68.75	−.03	624.36	4.870	54.440	1.0730	72.769	.000	−.010	
1806	.0002	20.779	115.018	68.71	68.79	−.12	701.10	4.473	55.130	1.4790	72.773	.000	−.000	
402	.0002	20.751	115.760	68.82	68.81	.02	750.67	4.874	55.410	1.4326	72.775	.000	−.000	
129	.0003	20.669	118.003	68.84	68.87	−.04	624.10	4.797	56.230	1.0730	72.780	.000	−.010	
1807	.0003	20.615	119.454	68.81	68.91	−.13	698.71	4.408	56.740	1.4790	72.784	.000	−.000	
403	.0004	20.573	120.599	68.93	68.94	−.01	753.25	4.832	57.140	1.4326	72.786	.000	−.000	
130	.0005	20.493	122.751	68.97	69.00	−.04	623.42	4.727	57.870	1.0730	72.792	.000	−.020	
1808	.0006	20.455	123.782	68.95	69.03	−.11	681.47	4.254	58.210	1.4790	72.794	.000	.000	
404	.0006	20.438	124.252	68.99	69.04	−.07	755.80	4.808	58.370	1.4326	72.795	.000	−.000	
131	.0009	20.320	127.423	69.06	69.14	−.11	620.83	4.650	59.380	1.0730	72.803	.000	−.030	
405	.0011	20.261	129.007	69.22	69.19	.04	754.44	4.744	59.870	1.4326	72.807	.000	−.000	
132	.0016	20.148	132.037	69.29	69.30	−.01	624.02	4.617	60.770	1.0730	72.815	.000	−.040	
406	.0020	20.085	133.712	69.39	69.36	.05	754.17	4.694	61.250	1.4326	72.819	.000	−.000	
133	.0026	19.985	136.389	69.50	69.46	.05	686.43	5.024	62.000	1.0730	72.826	.000	−.060	
407	.0032	19.911	138.362	69.62	69.55	.11	753.09	4.641	62.530	1.4326	72.832	.000	.000	
134	.0044	19.797	141.365	69.64	69.68	−.06	686.18	4.967	63.320	1.0730	72.840	.000	−.080	
412	.0049	19.757	142.426	69.79	69.73	.07	747.04	4.567	63.590	1.4326	72.842	.000	.010	
421	.0049	19.750	142.604	69.78	69.74	.05	690.48	4.221	63.630	1.4325	72.843	.000	.010	
135	.0070	19.611	146.274	69.98	69.93	.07	685.20	4.903	64.540	1.0730	72.853	.001	−.110	
501	.0090	19.499	149.205	70.13	70.09	.06	749.49	4.626	65.230	1.3802	72.861	.000	−.000	
136	.0106	19.425	151.131	70.22	70.21	.02	685.67	4.857	65.670	1.0730	72.866	.001	−.140	
502	.0133	19.323	153.786	70.37	70.37	.01	749.13	4.585	66.250	1.3802	72.873	.001	.010	
137	.0157	19.240	155.932	70.57	70.51	.08	684.57	4.800	66.710	1.0730	72.879	.001	−.180	
503	.0189	19.147	158.321	70.76	70.68	.11	748.66	4.541	67.210	1.3802	72.886	.001	.020	
138	.0226	19.055	160.684	70.84	70.85	−.01	684.78	4.757	67.690	1.0730	72.893	.001	−.220	
601	.0228	19.049	160.830	70.87	70.86	.01	751.08	4.670	67.720	1.3135	72.893	.001	−.020	
504	.0244	19.012	161.771	71.03	70.93	.15	398.68	2.403	67.910	1.3802	72.896	.001	.030	
518	.0254	18.992	162.290	70.95	70.97	−.03	463.12	2.792	68.010	1.3802	72.897	.001	.030	
505	.0259	18.981	162.575	70.96	70.99	−.05	307.56	1.853	68.070	1.3802	72.898	.001	.030	
139	.0315	18.870	165.384	71.22	71.21	.01	684.22	4.709	68.610	1.0730	72.906	.002	−.260	
602	.0317	18.867	165.464	71.23	71.22	.02	755.36	4.657	68.620	1.3135	72.907	.001	−.010	
140	0.0430	18.685	170.038	71.72	71.61	0.15	683.91	4.660	69.470	1.0730	72.920	0.002	−0.300	
603	.0431	18.684	170.061	71.69	71.61	.10	752.92	4.602	69.470	1.3135	72.920	.002	.010	
141	.0501	18.589	172.446	71.91	71.83	.11	758.69	5.147	69.890	1.0730	72.927	.003	−.320	
604	.0573	18.501	174.613	72.08	72.04	.06	752.45	4.563	70.260	1.3135	72.933	.002	.030	
142	.0683	18.382	177.535	72.34	72.33	.02	758.05	5.096	70.750	1.0730	72.942	.004	−.360	
605	.0749	18.317	179.126	72.52	72.49	.03	751.80	4.524	71.010	1.3135	72.947	.003	.060	
143	.0909	18.176	182.564	72.90	72.87	.04	756.20	5.034	71.560	1.0730	72.957	.005	−.400	
606	.0963	18.133	183.603	73.00	72.98	.03	753.08	4.497	71.720	1.3135	72.960	.004	.110	
701	.1119	18.016	186.415	73.42	73.31	.15	755.67	4.638	72.150	1.2363	72.969	.005	−.020	
144	.1187	17.968	187.540	73.46	73.45	.02	756.57	4.990	72.320	1.0730	72.972	.006	−.430	
101	.1200	17.960	187.745	73.37	73.47	−.13	739.12	4.877	72.350	1.0730	72.973	.006	−.430	
702	.1416	17.822	190.995	73.94	73.88	.08	754.21	4.593	72.820	1.2363	72.982	.006	.030	
102	.1530	17.756	192.556	74.06	74.08	−.02	738.00	4.824	73.040	1.0730	72.987	.007	−.440	
703	.1767	17.628	195.533	74.49	74.49	.00	753.06	4.552	73.450	1.2363	72.997	.007	.090	
103	.1923	17.550	197.332	74.75	74.74	.01	740.70	4.798	73.690	1.0730	73.002	.009	−.450	
704	.2177	17.432	200.029	75.14	75.13	.01	753.12	4.517	74.050	1.2363	73.011	.008	.170	
104	.2383	17.342	202.057	75.40	75.44	−.05	735.51	4.724	74.310	1.0730	73.017	.010	−.430	
301	.2391	17.339	202.127	75.45	75.45	−.00	746.93	4.571	74.320	1.1811	73.017	.009	.020	
705	.2651	17.234	204.490	75.90	75.83	.09	752.84	4.478	74.620	1.2363	73.025	.010	.270	
302	.2694	17.217	204.866	75.76	75.89	−.17	748.89	4.565	74.660	1.1811	73.026	.010	.070	
105	.2915	17.133	206.723	76.15	76.19	−.05	735.14	4.681	74.890	1.0730	73.032	.012	−.390	
706	.3186	17.036	208.850	76.43	76.55	−.16	728.54	4.307	75.150	1.2363	73.039	.011	.400	
303	.3255	17.012	209.372	76.56	76.64	−.11	748.35	4.523	75.210	1.1811	73.040	.012	.180	
106	.3529	16.921	211.363	76.97	77.00	−.04	740.46	4.675	75.450	1.0730	73.047	.014	−.330	
304	.3893	16.806	213.835	77.41	77.45	−.06	747.19	4.480	75.730	1.1811	73.055	.014	.320	
107	.4230	16.705	215.978	77.87	77.86	.01	739.54	4.628	75.980	1.0730	73.062	.017	−.230	
305	.4613	16.596	218.259	78.37	78.32	.07	746.39	4.437	76.230	1.1811	73.069	.016	.490	
108	.5020	16.487	220.536	78.87	78.79	.09	735.37	4.561	76.480	1.0730	73.077	.019	−.100	
306	.5421	16.384	222.647	79.13	79.25	−.14	745.84	4.404	76.700	1.1811	73.084	.019	.700	
109	.5907	16.264	225.058	79.79	79.79	−.00	738.77	4.546	76.960	1.0730	73.092	.022	.070	
110	.6896	16.038	229.541	80.79	80.86	−.09	734.29	4.483	77.410	1.0730	73.107	.025	.300	
801	.7366	15.937	231.507	81.41	81.36	.05	769.10	4.614	77.610	1.1028	73.114	.026	.670	
111	.7992	15.807	233.988	81.97	82.03	−.07	739.19	4.474	77.850	1.0730	73.122	.029	.580	
802	.8553	15.695	236.091	82.60	82.61	−.01	773.02	4.600	78.050	1.1028	73.129	.029	1.010	
112	.9344	15.543	238.894	83.41	83.44	−.03	810.00	4.856	78.310	1.0730	73.139	.033	.970	
803	.9863	15.447	240.641	84.06	83.97	.10	772.07	4.551	78.480	1.1028	73.145	.033	1.430	
113	1.0825	15.275	243.704	84.90	84.97	−.09	809.41	4.809	78.750	1.0730	73.156	.038	1.460	
804	1.1189	15.212	244.814	85.26	85.35	−.11	655.14	3.836	78.850	1.1028	73.160	.037	1.910	
901	1.2188	15.043	247.726	86.41	86.40	.02	786.22	4.725	79.110	1.0260	73.171	.043	1.380	
114	1.2453	14.999	248.469	86.56	86.67	−.13	808.37	4.758	79.180	1.0730	73.173	.043	2.070	
902	1.3930	14.761	252.414	88.29	88.24	.05	786.10	4.678	79.520	1.0260	73.188	.048	2.060	
903	1.5827	14.468	257.059	90.39	90.33	.07	784.89	4.625	79.900	1.0260	73.205	.054	2.910	
1001	1.7130	14.274	260.020	91.87	91.81	.06	785.82	4.755	80.150	.9495	73.216	.063	2.270	
905	1.7461	14.225	260.744	92.04	92.19	−.17	787.80	4.611	80.200	1.0260	73.218	.060	3.740	
904	1.7756	14.182	261.383	92.32	92.54	−.24	687.69	4.021	80.250	1.0260	73.221	.061	3.910	
1002	1.9373	13.948	264.754	94.60	94.50	.11	785.32	4.699	80.520	.9495	73.234	.071	3.450	
1003	2.1805	13.603	269.456	97.75	97.66	.09	790.15	4.675	80.890	.9495	73.252	.080	5.000	
1004	2.4435	13.236	274.128	101.37	101.47	−.10	789.86	4.622	81.240	.9494	73.270	.089	7.050	
1101	2.4671	13.203	274.528	101.81	101.84	−.03	784.34	4.725	81.270	.8786	73.272	.097	5.490	0.002
1102	2.7585	12.795	279.261	106.68	106.78	−.09	783.94	4.661	81.620	.8786	73.290	.108	8.260	.005
1401	3.0487	12.381	283.620	112.75	112.69	.05	753.66	4.841	81.940	.6739	73.308	.156	3.470	.013
1103	3.0713	12.348	283.946	112.85	113.21	−.32	784.57	4.602	81.960	.8785	73.309	.121	12.230	.012
1402	3.3806	11.888	288.238	121.72	121.33	.32	659.46	4.154	82.260	.6739	73.327	.174	8.440	.022
1601	3.6532	11.456	291.779	130.47	130.84	−.29	749.65	4.389	82.510	.8108	73.342	.158	21.760	.048
1403	3.7091	11.364	292.479	133.41	133.19	.16	662.09	4.093	82.560	.6739	73.345	.193	16.110	.048
1414	3.7139	11.355	292.539	133.54	133.40	.11	651.15	4.025	82.560	.6739	73.345	.193	16.250	.047
1701	3.9357	10.967	295.236	146.32	144.80	1.04	496.69	3.142	82.750	.5795	73.357	.240	16.650	.062
1415	4.0476	10.756	296.550	151.71	152.27	−.37	651.54	3.944	82.840	.6738	73.363	.213	29.830	.143
1404	4.0571	10.737	296.661	152.77	152.98	−.14	661.25	3.994	82.840	.6738	73.363	.213	30.370	.146
1702	4.2086	10.426	298.389	167.24	166.28	.57	499.15	3.091	82.960	.5795	73.371	.259	32.170	.153
1416	4.3812	10.025	300.295	187.54	188.77	−.66	589.30	3.467	83.090	.6738	73.379	.232	59.180	.331
1405	4.4048	9.965	300.550	191.34	192.82	−.77	590.93	3.471	83.110	.6738	73.380	.234	62.640	.366
1703	4.4911	9.729	301.473	212.29	210.92	.64	499.06	2.990	83.170	.5794	73.384	.278	68.370	.358

**Table 5:** Comparison of the calculated C_σ_ with results of others

Temperature *K*	*C_σ_*-calculated this paper J/mol-K	*C*_0_ from the literature J/mol-K	Difference in 0/0

Reference [16]			

96.77	68.27	68.46	−0.3
96.82	68.28	68.76	−.7
98.06	68.32	68.55	−.3
101.54	68.44	68.71	−.4
107.08	68.60	68.63	−.0
108.65	68.64	68.55	.1
115.74	68.81	68.67	.2
116.19	68.82	68.45	.5
122.70	69.00	69.13	−.2
123.60	69.02	69.17	−.2
128.08	69.16	69.55	−.6
128.49	69.17	69.51	−.5
132.65	69.32	69.84	−.8
138.00	69.53	69.89	−.5
138.18	69.54	70.01	−.7
138.31	69.55	69.84	−.4
142.43	69.73	70.10	−.5
143.36	69.78	70.05	−.4
151.75	70.24	69.97	.4
152.60	70.30	70.14	.2
154.99	70.45	70.26	.3
156.98	70.58	70.26	.5
157.42	70.61	70.10	.7
160.10	70.80	71.10	−.4
162.65	70.99	71.18	−.3
164.49	71.14	71.73	−.8
165.93	71.26	71.31	−.1
168.09	71.44	71.60	−.2
170.18	71.62	71.77	−.2
172.05	71.79	71.77	.0
172.69	71.85	72.15	−.4
178.17	72.39	72.82	−.6
181.50	72.75	73.07	−.4
182.03	72.81	73.32	−.7
190.00	73.75	73.53	.3
199.86	75.11	74.37	1.0
208.88	76.56	75.66	1.2
212.80	77.26	75.96	1.7
220.48	78.78	77.80	1.2
228.76	80.67	80.61	.1
236.21	82.65	82.32	.4
244.61	85.28	83.96	1.6
252.53	88.29	87.14	1.3
258.22	90.89	88.44	2.7
265.25	94.80	92.33	2.6
273.06	100.53	98.11	2.4
278.07	105.41	101.33	3.9
284.07	113.41	109.12	3.8
291.27	129.25	122.69	5.1
294.85	142.89	135.79	5.0

Reference [17]			

91.59	68.03	68.30	−0.4
92.97	68.10	68.46	−.5
94.94	68.20	68.38	−.3
96.60	68.27	68.25	.0
98.23	68.33	68.88	−.8
98.89	68.35	68.38	−.0
100.49	68.41	68.34	.1
104.05	68.51	68.59	−.1
106.67	68.59	68.88	−.4
109.24	68.65	68.92	−.4
111.67	68.71	69.13	−.6
114.20	68.77	69.01	−.3
116.24	68.82	69.17	−.5
119.33	69.90	69.30	−.6
122.72	69.00	69.43	−.6
125.96	69.09	69.59	−.7
129.47	69.20	69.55	−.5
134.49	69.39	69.64	−.4
138.72	69.56	69.76	−.3
142.83	69.75	69.89	−.2
145.97	69.91	70.05	−.2
149.80	70.13	70.31	−.3
153.58	70.36	70.77	−.6
157.95	70.65	70.77	−.2
162.82	71.01	71.02	−.0
167.43	71.38	71.52	−.2
172.02	71.79	71.56	.3
176.54	72.23	72.06	.2
180.88	72.68	72.11	.8

**Table 6:** The specific heat, *Cv*, of singlephase fluid ethane

Run No. point No.	PVT conditions			*c_v_* J/mol-K	*C_v_* calc. from Goodwin [2] J/mol-K	Dev. 0/0	Heat, *Q J*	Δ*T* *K*	*C_o_ J*/*K*	Sample mol	Calorimeter volume cm^3^	Volume change correction J/mol-K
	Pressure MPa	Density mol/l	Temperature *K*									
												
116	4.1720	14.644	258.973	47.20	47.10	0.20	806.66	6.065	80.060	1.0725	73.240	2.173
117	7.5074	14.628	265.038	47.57	47.58	−.03	805.26	6.054	80.550	1.0722	73.297	1.372
118	10.8447	14.616	271.085	48.17	48.10	.14	806.72	6.015	81, 010	1.0721	73.354	1.371
119	14.1443	14.604	277.105	48.61	48.64	−.07	805.29	5.962	81.450	1.0721	73.412	1.386
120	17.4012	14.592	283.089	49.12	49.21	−.18	805.78	5.922	81.900	1.0721	73.470	1.407
121	20.6171	14.580	289.038	49.76	49.79	−.06	804.40	5.863	82.320	1.0721	73.528	1.429
122	21.1487	14.578	290.026	49.84	49.89	−.09	806.75	5.873	82.390	1.0721	73.538	1.433
123	24.3353	14.567	295.968	50.48	50.48	−.01	806.34	5.823	82.800	1.0720	73.596	1.456
124	27.4843	14.555	301.879	51.13	51.09	.06	805.76	5.772	83.190	1.0720	73.654	1.480
125	30.6035	14.543	307.773	51.82	51.71	.22	806.49	5.730	83.580	1.0720	73.712	1.503
126	33.6956	14.532	313.655	52.46	52.32	.25	805.85	5.682	83.960	1.0720	73.770	1.527
146	3.3933	14.647	257.577	47.20	47.00	.42	404.93	3.029	79.950	1.0726	73.226	2.898
147	5.0544	14.637	260.618	47.30	47.23	.16	404.05	3.050	80.190	1.0722	73.255	1.438
148	6.7546	14.631	263.680	47.38	47.47	−.19	405.73	3.057	80.440	1.0722	73.284	1.384
149	8.4463	14.624	266.736	47.74	47.73	.02	404.93	3.037	80.680	1.0722	73.313	1.375
150	10.1277	14.618	269.783	47.92	47.99	−.13	404.36	3.023	80.910	1.0721	73.342	1.376
151	11.7992	14.612	272.823	48.20	48.25	−.10	404.37	3.011	81.140	1.0721	73.371	1.382
152	13.4607	14.606	275.855	48.48	48.53	−.10	403.76	2.994	81.370	1.0721	73.400	1.390
208	3.6415	20.996	110.494	44.79	45.02	−.52	249.72	1.938	53.390	1.5284	72.793	4.605
209	7.4628	20.981	112.401	46.07	45.06	2.18	245.90	1.896	54.140	1.5280	72.829	3.385
210	11.4471	20.969	114.288	46.20	45.10	2.39	247.71	1.897	54.860	1.5279	72.868	3.332
211	15.3828	20.957	116.167	46.21	45.13	2.33	246.42	1.878	55.560	1.5279	72.906	3.325
212	19.8576	20.944	118.324	46.28	45.17	2.41	315.54	2.464	56.340	1.5279	72.950	3.323
213	24.8511	20.930	120.762	46.41	45.20	2.62	324.08	2.433	57.200	1.5279	73.000	3.338
214	29.7379	20.915	123.178	46.43	45.22	2.60	324.68	2.422	58.010	1.5278	73.050	3.356
308	3.6029	16.140	231.182	45.31	44.84	1.03	402.58	2.983	77.580	1.1806	73.143	3.292
309	5.8108	16.129	234.160	45.16	45.07	.21	404.39	3.032	77.870	1.1802	73.176	1.870
310	8.0795	16.121	237.158	45.53	45.30	.50	405.00	3.022	78.150	1.1802	73.211	1.823
311	10.3326	16.113	240.148	45.79	45.53	.56	404.52	3.005	78.430	1.1802	73.245	1.815
312	12.5647	16.105	243.126	46.06	45.77	.63	404.88	2.995	78.700	1.1801	73.279	1.818
313	13.6122	16.101	244.528	46.08	45.88	.44	806.74	5.961	78.830	1.1801	73.295	1.811
314	17.9966	16.086	250.439	46.68	46.35	.71	806.99	5.908	79.350	1.1801	73.363	1.830
315	22.3017	16.071	256.303	47.34	46.84	1.06	806.20	5.847	79.840	1.1801	73.431	1.854
316	26.5329	16.056	262.126	47.92	47.91	.01	806.48	5.799	80.310	1.1801	73.499	1.880
317	30.7076	16.041	267.928	48.53	48.36	.35	806.20	5.747	80.770	1.1800	73.566	1.907
318	33.7881	16.029	272.246	48.91	48.72	.39	402.54	2.853	81.100	1.1800	73.617	1.937
409	1.6032	19.657	145.929	44.78	43.67	2.49	161.75	1.212	64.450	1.4324	72.866	3.424
414	4.7513	19.643	148.039	44.89	43.73	2.57	421.18	3.121	64.950	1.4320	72.901	4.002
410	4.7903	19.643	148.064	44.98	43.74	2.76	415.86	3.078	64.960	1.4320	72.902	4.013
411	9.4211	19.624	151.130	44.41	43.83	1.20	415.62	3.113	65.670	1.4316	72.953	2.975
415	9.4536	19.624	151.151	44.39	43.83	1.25	420.32	3.149	65.670	1.4316	72.954	2.974
416	14.2813	19.609	154.270	44.48	43.93	1.24	421.01	3.135	66.360	1.4316	73.008	2.957
417	19.0188	19.594	157.366	44.66	44.03	1.41	420.11	3.107	67.020	1.4316	73.062	2.965
418	23.6646	19.579	160.437	44.84	44.13	1.58	419.85	3.085	67.640	1.4315	73.115	2.980
419	28.2240	19.565	163.485	44.93	44.23	1.57	420.06	3.069	68.250	1.4315	73.169	2.999
420	31.7899	19.553	165.894	45.10	44.30	1.76	248.70	1.807	68.700	1.4315	73.211	3.026
423	4.7430	19.643	148.027	44.78	43.73	2.33	424.39	3.155	64.950	1.4320	72.901	3.802
424	9.4819	19.624	151.152	44.52	43.83	1.54	421.19	3.147	65.670	1.4317	72.954	3.103
425	14.2777	19.609	154.267	44.55	43.93	1.39	420.94	3.132	66.350	1.4316	73.008	2.989
426	19.0103	19.594	157.364	44.65	44.03	1.40	420.28	3.108	67.020	1.4315	73.062	2.981
509	14.2771	18.877	174.677	44.83	43.83	2.23	434.68	3.198	70.280	1.3793	73.072	2.761
511	3.8855	18.916	166.750	44.64	43.48	2.59	443.61	3.280	68.860	1.3798	72.947	3.460
512	8.1541	18.898	170.023	44.52	43.62	2.02	444.61	3.289	69.460	1.3795	72.998	3.104
513	12.4645	18.883	173.301	44.48	43.77	1.60	444.40	3.287	70.040	1.3794	73.050	2.774
514	16.7633	18.868	176.572	44.50	43.91	1.32	444.52	3.274	70.590	1.3793	73.102	2.754
515	20.9940	18.855	179.822	44.71	44.06	1.47	443.92	3.250	71.130	1.3793	73.154	2.759
516	25.1643	18.841	183.059	44.81	44.20	1.35	444.90	3.214	71.640	1.3793	73.206	2.771
517	29.2726	18.827	186.280	45.01	44.35	1.47	444.92	3.223	72.130	1.3792	73.257	2.788
520	3.2642	18.919	166.260	44.15	43.46	1.57	445.77	3.323	68.770	1.3799	72.940	3.219
507	5.8435	18.908	168.242	44.19	43.55	1.45	435.73	3.226	69.140	1.3797	92.970	3.594
521	7.5377	18.901	169.533	44.21	43.60	1.37	442.92	3.280	69.370	1.3796	72.990	3.382
522	11.7968	18.885	172.795	44.17	43.74	.96	444.97	3.303	69.950	1.3794	73.042	2.783
523	16.0901	18.871	176.058	44.27	43.89	.86	444.69	3.285	70.510	1.3793	73.094	2.755
524	20.3225	18.857	179.304	44.43	44.03	.90	445.20	3.270	71.040	1.3793	73.145	2.757
525	24.4871	18.843	182.531	44.63	44.18	1.00	444.17	3.244	71.550	1.3793	73.197	2.769
526	28.5822	18.830	185.736	44.83	44.32	1.12	445.21	3.233	72.050	1.3792	73.249	2.785
527	32.6165	18.816	188.925	45.12	44.47	1.45	444.58	3.207	72.520	1.3792	73.300	2.802
608	2.6873	17.990	189.118	44.55	43.56	2.22	442.40	3.284	72.550	1, 3133	73.002	2.800
609	6.1932	17, 974	192.360	44.21	43.74	1.06	444.14	3.281	73.010	1.3130	73.047	3.285
610	9.6987	17.959	195.613	44.09	43.93	.37	444.20	3.299	73.460	1.3127	73.093	2.520
611	12.3412	17.950	198.035	44.27	44.07	.47	444.48	3.289	73.790	1.3127	73.127	2.463
612	15.8638	17.938	201.281	44.50	44.25	.55	446.15	3.285	74.210	1.3126	73.174	2.448
613	19.3395	17.927	204.510	44.71	44.44	.59	442.66	3.242	74.620	1.3126	73.220	2.452
614	22.7639	17.915	207.717	44.94	44.63	.69	444.78	3.241	75.010	1.3126	73.266	2.462
615	26.1418	17.903	210.907	45.21	44.81	.87	442.70	3.208	75.390	1.3125	73.312	2.476
616	29.4745	17.892	214.081	45.46	45.00	1.01	444.44	3.204	75.760	1.3125	73.358	2.481
617	32.7619	17.881	217.237	45.74	45.19	1.21	442.86	3.176	76.120	1.3125	73.403	2.508
708	2.9578	16.914	214.213	44.89	44.16	1.63	526.64	3.923	75.780	1.2361	73.083	2.402
709	6.3377	16.898	218.081	44.88	44.42	1.02	524.81	3.882	76.120	1.2358	73.130	2.834
710	9.6894	16.884	221.935	44.97	44.69	.62	524.91	3.893	76.630	1.2355	73.178	2.141
711	13.0632	16.872	225.787	45.16	44.96	.44	525.14	3.878	77.030	1.2355	73.226	2.092
712	15.3575	16.864	228.421	45.31	45.15	.36	526.13	3.873	77.300	1.2354	73.259	2.088
713	18.6724	16.852	232.253	45.70	45.42	.62	525.64	3.844	77.680	1.2354	73.308	2.095
714	21.9409	16.841	236.060	45.98	45.69	.63	525.92	3.826	78.050	1.2354	73.356	2.107
715	25.1669	16.830	239.848	46.36	45.96	.86	525.73	3.801	78.400	1.2354	73.404	2.122
716	28.3479	16.818	243.612	46.75	46.24	1.09	524.19	3.767	78.750	1.2353	73.452	2.139
717	31.4873	16.807	247.355	47.07	46.52	1.17	525.11	3.754	79.080	1.2353	73.500	2.156
806	3.1815	15.063	250.496	46.73	45.87	1.84	530.46	3.995	79.350	1.1026	73.201	1.747
807	5.5686	15.051	254.472	46.78	46.21	1.22	533.28	3.987	79.690	1.1023	73.240	2.252
808	7.9440	15.039	258.449	47.15	47.19	−.09	532.66	3.982	80.020	1.1021	73.280	1.623
809	10.3307	15.030	262.425	47.46	47.50	−.08	532.93	3.968	80.340	1.1020	73.319	1.531
810	6.5052	15.046	256.033	46.91	46.35	1.19	534.05	3.989	79.820	1.1022	73.256	2.135
811	8.8884	15.035	260.021	47.20	47.31	−.24	533.40	3.984	80.150	1.1020	73.295	1.562
812	11.2775	15.027	264.005	47.52	47.62	−.22	533.80	3.969	80.460	1.1020	73.335	1.523
813	13.6450	15.018	267.971	47.97	47.95	.05	533.07	3.940	80.770	1.1019	73.375	1.519
814	15.9881	15.009	271.915	48.29	48.29	.01	532.91	3.919	81.080	1.1019	73.415	1.526
815	18.3110	15.001	275.845	48.66	48.64	.03	532.27	3.894	81.370	1.1019	73.455	1.536
816	20.6178	14.993	279.768	49.05	49.00	.10	532.98	3.879	81.660	1.1019	73.495	1.549
817	22.9060	14.984	283.679	49.41	49.37	.08	532.59	3.856	81.940	1.1019	73.535	1.563
818	25.1761	14.976	287.579	49.75	49.75	−0	532.33	3.836	82.220	1.1019	73.575	1.578
819	27.4251	14.968	291.462	50.27	50.13	.28	532.26	3.812	82.490	1.1018	73.615	1.593
820	29.6660	14.959	295.349	50.62	50.52	.20	531.12	3.785	82.750	1.1018	73.655	1.609
821	32.3464	14.949	300.025	51.18	51.00	.36	760.20	5.382	83.070	1.1018	73.703	1.624
907	3.3507	14.004	266.868	48.30	47.62	1.42	533.44	4.050	80.690	1.0259	73.256	1.430
908	5.2994	13.994	270.918	48.29	47.97	.66	532.42	4.019	81.000	1.0256	73.292	1.893
909	7.2349	13.983	274.958	48.61	48.33	.56	532.78	4.019	81.300	1.0454	73.327	1.401
910	9.1760	13.976	278.996	48.89	48.71	.38	532.34	4.002	81.600	1.0253	73.362	1.259
911	11.1122	13.968	283.030	49.18	49.09	.18	532.86	3.989	81.890	1.0252	73.398	1.242
912	13.0368	13.961	287.053	49.57	49.49	.17	532.29	3.964	82.180	1.0252	73.434	1.232
913	14.9508	13.954	291.068	49.99	49.88	.22	532.31	3.943	82.460	1.0252	73.470	1.250
914	16.8538	13.947	295.074	50.34	50.29	.10	532.39	3.925	82.740	1.0252	73.506	1.260
915	18.7466	13.940	299.072	50.72	50.70	.04	532.40	3.906	83.010	1.0252	73.542	1.272
916	20.6314	13.933	303.068	51.12	51.11	.02	532.51	3.887	83.270	1.0251	73.578	1.285
917	22.5043	13.926	307.053	51.58	51.53	.10	530.25	3.850	83.540	1.0251	73.614	1.299
919	24.5909	13.918	311.509	52.00	52.00	0	530.82	3.833	83.820	1.0251	73.655	1.315
920	26.4506	13.911	315.497	52.44	52.42	.05	530.30	3.810	84.080	1.0251	73.691	1.329
921	28.3059	13.904	319.489	52.84	52.84	.01	531.84	3.802	84.330	1.0251	73.728	1.343
922	30.1546	13.897	323.481	53.30	53.26	.07	529.10	3.763	84.580	1.0251	73.764	1.358
923	31.9987	13.890	327.478	53.80	53.68	.21	530.45	3.752	84.830	1.0251	73.801	1.372
1006	3.8940	12.950	280.688	49.94	48.89	2.11	532.04	4.084	81.730	0.9493	73.308	1.183
1007	5.4845	12.941	284.835	49.93	49.29	1.29	531.99	4.063	82.020	.9491	73.340	1.602
1008	7.0695	12.932	288.976	50.22	49.69	1.06	534.04	4.075	82.310	.9488	73.372	1.136
1009	8.6596	12.925	293.117	50.47	50.09	.75	532.16	4.048	82.600	.9488	73.404	1.024
1010	8.7885	12.925	293.453	50.60	50.13	.94	534.37	4.061	82.620	.9488	73.407	1.021
1011	10.3808	12.918	297.602	50.83	50.54	.57	533.14	4.036	82.910	.9487	73.440	1.008
1012	11.9663	12.912	301.740	51.20	50.96	.46	532.61	4.013	83.180	.9487	73.472	1.009
1014	15.1274	12.900	310.014	51.71	51.81	−.20	533.64	3.990	83.730	.9487	73.538	1.026
1015	16.7073	12.894	314.163	52.22	52.25	−.05	531.89	3.954	83.990	.9486	73.571	1.037
1016	18.2826	12.888	318.308	52.68	52.68	0	531.33	3.929	84.260	.9486	73.605	1.049
1017	19.8541	12.882	322.453	53.17	53.11	.11	532.03	3.913	84.520	.9486	73.638	1.061
1018	21.4289	12.876	326.617	53.56	53.55	.03	532.16	3.896	84.780	.9486	73.672	1.074
1105	4.3795	11.976	290.820	51.74	50.15	3.07	536.23	4.164	82.440	.8784	73.347	1.012
1106	5.6995	11.968	295.076	51.64	50.54	2.13	336.58	4.150	82.740	.8782	73.377	1.372
1107	4.5546	11.975	291.384	51.62	50.20	2.75	538.51	4.183	82.480	.8784	73.351	1.038
1108	5.8805	11.967	295.658	51.73	50.60	2.19	537.49	4.154	82.780	.8781	73.381	1.346
1109	7.2048	11.959	299.925	51.98	51.00	1.88	532.89	4.116	83.060	.8779	73.411	0.890
1110	8.5348	11.954	304.195	52.14	51.42	1.39	537.27	4.137	83.350	.8779	73.441	.838
1111	9.8732	11.948	308.490	52.37	51.84	1.02	537.32	4.123	83.630	.8779	73.471	.829
1112	11.1743	11.943	312.666	52.65	52.26	.76	504.24	3.854	83.900	.8778	73.501	.832
1113	12.4352	11.938	316.715	52.99	52.66	.62	502.63	3.825	84.160	.8778	73.530	.839
1114	13.6959	11.933	320.765	53.33	53.07	.49	501.18	3.798	84.410	.8778	73.559	.847
1115	14.9594	11.928	324.828	53.62	53.48	.27	503.47	3.800	84.670	.8778	73.588	.857
1116	16.2269	11.923	328.907	54.03	53.89	.26	502.60	3.776	84.920	.8778	73.618	.867
1201	5.1530	5.887	308.511	68.85	61.64	10.47	501.98	4.420	83.630	.4322	73.416	.435
1202	5.5670	5.884	313.190	62.98	59.72	5.17	503.22	4.518	83.930	.4321	73.436	.572
1203	5.9863	5.881	317.960	60.78	58.79	3.27	503.13	4.544	84.240	.4320	73.457	.538
1204	6.4088	5.878	322.792	59.47	58.27	2.02	502.67	4.553	84.540	.4319	73.479	.412
1205	6.8341	5.876	327.675	58.71	57.97	1.26	502.69	4.555	84.840	.4319	73.501	.366
1301	4.9833	4.611	308.116	62.03	58.83	5.16	426.37	4.071	83.600	.3385	73.412	.365
1302	5.2602	4.609	312.344	59.59	57.95	2.76	410.37	3.939	83.880	.3384	73.430	.427
1303	5.5228	4.607	316.397	58.61	57.45	1.97	380.37	3.652	84.140	.3383	73.447	.545
1304	5.7766	4.604	320.349	57.30	57.15	.27	375.90	3.616	84.390	.3383	73.463	.533
1305	6.0260	4.603	324.259	56.95	56.97	−.02	377.80	3.631	84.630	.3382	73.479	.428
1306	6.2766	4.601	328.210	56.92	56.88	.08	378.42	3.630	84.880	.3381	73.496	.363
1407	5.2172	9.177	306.501	59.73	53.88	9.79	377.40	3.038	83.500	.6737	73.410	.728
1408	5.7778	9.173	309.793	58.44	53.88	7.80	375.80	3.038	83.710	.6735	73.427	.899
1409	6.3472	9.169	313.122	57.70	53.99	6.44	375.87	3.051	83.930	.6734	73.445	.623
1410	6.9235	9.166	316.478	57.34	54.15	5.57	376.10	3.055	84.140	.6733	73.464	.539
1413	8.6765	9.158	326.629	56.92	54.81	3.70	373.93	3.029	84.780	.6733	73.519	.505
1418	5.1849	9.177	306.311	59.88	53.88	10.01	373.34	3.003	83.490	.6737	73.409	.714
1419	5.7106	9.173	309.399	58.49	53.87	7.89	371.75	3.005	83.690	.6735	73.425	.915
1420	6.2429	9.169	312.513	57.77	53.96	6.59	371.21	3.012	83.890	.6734	73.442	.659
1421	6.7799	9.166	315.643	57.36	54.11	5.67	372.18	3.024	84.090	.6733	73.459	.550
1422	7.3256	9.164	318.812	57.04	54.28	4.82	372.31	3.026	84.290	.6733	73.476	.520
1423	7.8739	9.161	321.990	56.95	54.49	4.33	372.44	3.024	84.490	.6733	73.494	.509
1424	8.4266	9.159	325.186	56.77	54.71	3.62	371.33	3.013	84.690	.6733	73.511	.506
1425	8.9849	9.156	328.408	56.85	54.94	3.34	372.69	3.018	84.890	.6733	73.529	.506
1501	4.1439	3.276	298.669	60.31	55.73	7.58	372.96	3.824	82.980	0.2404	73.371	0.260
1502	4.3292	3.276	302.904	56.64	55.19	2.56	435.15	4.489	83.260	.2404	73.387	.264
1503	4.5274	3.275	307.501	55.60	54.85	1.36	436.31	4.498	83.560	.2404	73.405	.271
1504	4.7242	3.274	312.122	55.11	54.66	.81	435.24	4.479	83.860	.2404	73.423	.281
1505	4.9200	3.272	316.773	54.33	54.59	−.48	437.11	4.493	84.160	.2403	73.441	.296
1506	5.1138	3.271	321.425	53.93	54.62	−1.28	434.99	4.462	84.450	.2403	73.459	.321
1507	5.3073	3.270	326.113	53.91	54.71	−1.48	436.78	4.467	84.750	.2403	73.477	.366
1603	4.6182	11.049	297.554	53.79	51.31	4.60	375.57	2.952	82.900	.8107	73.373	.860
1604	5.3719	11.044	300.530	53.36	51.55	3.39	372.72	2.927	83.100	.8105	73.391	1.186
1605	6.1267	11.039	303.509	53.51	51.80	3.20	374.13	2.935	83.300	.8104	73.410	1.007
1606	6.8874	11.035	306.501	53.59	52.06	2.85	373.14	2.926	83.500	.8103	73.429	.757
1607	7.6523	11.031	309.501	53.58	52.33	2.34	372.69	2.919	83.690	.8102	73.448	.712
1608	8.4210	11.028	312.511	53.55	52.60	1.76	372.65	2.915	83.890	.8102	73.468	.699
1609	9.1928	11.025	315.531	53.68	52.88	1.49	373.04	2.911	84.080	.8102	73.487	.695
1610	9.9703	11.021	318.570	53.85	53.16	1.27	373.46	2.907	84.270	.8102	73.507	.696
1611	10.7480	11.018	321.607	54.04	53.45	1.09	371.74	2.886	84.470	.8101	73.527	.699
1612	11.5291	11.015	324.656	54.14	53.74	.74	373.12	2.890	84.660	.8101	73.546	.704
1613	12.4082	11.012	328.087	54.44	54.06	.70	464.76	3.588	84.870	.8101	73.569	.709
1705	5.2078	7.891	307.853	67.13	55.71	17.02	374.85	3.052	83.590	.5793	73.414	.600
1706	5.6238	7.888	311.011	63.75	55.19	13.43	372.84	3.077	83.790	.5792	73.430	.788
1707	6.0466	7.884	314.211	62.20	55.05	11.49	373.21	3.101	84.000	.5791	73.445	.605
1708	6.4742	7.882	317.440	61.26	55.06	10.11	372.18	3.103	84.200	.5790	73.461	.481
1709	6.9054	7.880	320.690	60.54	55.15	8.91	372.05	3.108	84.410	.5790	73.478	.444
1710	6.9598	7.879	321.099	60.20	55.16	8.37	374.42	3.132	84.430	.5790	73.480	.442
1711	7.4171	7.877	324.541	59.88	55.31	7.63	409.73	3.427	84.650	.5789	73.497	.428
1712	7.8992	7.875	328.165	59.49	55.50	6.70	412.12	3.447	84.870	.5789	73.515	.423
1810	6.0623	20.289	131.088	45.99	44.25	3.77	381.55	2.837	60.490	1.4783	72.866	4.067
1811	11.0190	20.270	133.939	45.15	44.31	1.85	381.37	2.874	61.320	1.4780	72.918	3.151
1812	16.1127	20.254	136.801	45.23	44.37	1.89	382.21	2.861	62.110	1.4780	72.972	3.144
1813	21.1154	20.239	139.647	45.25	44.43	1.80	381.25	2.836	62.870	1.4780	73.025	3.155
1814	26.0147	20.224	142.469	45.40	44.49	2.01	381.83	2.820	63.600	1.4779	73.079	3.172
1815	30.8178	20.209	145.270	45.46	44.54	2.03	380.71	2.795	64.290	1.4779	73.132	3.192
1901	2.7144	1.585	288.868	49.30	48.60	1.43	280.23	3.182	82.310	.1162	73.322	.173
1902	2.7708	1.585	292.068	49.21	48.71	1.02	281.45	3.189	82.530	.1162	73.333	.174
1903	2.8271	1.585	295.282	48.28	48.84	−1.16	281.02	3.180	82.750	.1162	73.344	.175
1904	2.8980	1.585	299.364	48.87	49.04	−.34	435.51	4.908	83.030	.1162	73.359	.176
1905	2.9836	1.584	304.333	49.32	49.32	−0	436.70	4.901	83.360	.1162	73.376	.178
1906	3.0687	1.584	309.311	49.10	49.65	−1.10	435.44	4.870	83.680	.1162	73.394	.180
1907	3.1531	1.583	314.289	49.85	50.00	−.31	436.06	4.855	84.000	.1162	73.412	.182
1908	3.2372	1.583	319.287	50.21	50.39	−.37	435.39	4.828	84.320	.1162	73.429	.184
1909	3.3213	1.582	324.315	50.18	50.81	−1.26	435.80	4.816	84.630	.1162	73.447	.187
1910	3.4004	1.582	329.076	51.11	51.22	−.22	383.50	4.219	84.930	.1162	73.464	.189

**Table 7:** Inter comparison of C_v_ arid C_p_

Pressure MPa ref. [18]	Density mol/1 This paper	Temperature *K* from eq. ref. [2]	*c_v_* J/mol-K Interpol. this paper	*PVT* Contr. J/mol-K calc. ref. [2]	*C_p_* J/mol-K calc. col. 4, 5	*C_p_* J/mol-K Interpol. ref [18]	Diff pet
3.4474	14.647	257.677	47.19	44.44	91.64	90.43	−1.3
6.8948	14.630	263.928	47.41	41.87	89.28	88.29	−1.1
10.3421	14.617	270.176	48.00	39.64	87.64	87.15	−.6
13.7895	14.605	276.459	48.55	37.72	86.27	86.01	−.3
6.8948	20.983	112.105	45.94	23.41	69.35	68.58	−1.1
13.7895	20.962	115.407	46.19	23.17	69.36	68.34	−1.5
6.8948	16.125	235.582	45.31	34.72	80.03	79.59	−.6
13.7895	16.100	244.770	46.08	32.80	78.88	79.09	.3
1.7237	19.656	146.015	44.79	26.12	70.91	69.97	−1.4
6.8948	19.634	149.450	44.69	25.80	70.49	70.06	−.6
13.7895	19.611	153.944	44.46	25.39	69.85	70.00	.2
6.8948	18.904	169.041	44.22	27.18	71.40	71.43	0
13.7895	18.879	174.296	44.80	26.63	71.44	70.92	−.7
6.8948	17.971	193.014	44.16	29.20	73.36	74.08	1.0
13.7895	17.945	199.373	44.38	28.38	72.76	72.67	−.1
6.8948	10.896	218.724	44.89	32.10	76.99	76.45	−.7
13.7895	16.869	226.616	45.20	30.77	75.97	76.03	.1
3.4474	15.062	250.932	46.72	41.53	88.26	87.42	−1.0
6.8948	15.044	256.692	46.98	39.54	86.53	85.99	−.6
10.3421	15.030	262.445	47.46	37.78	85.24	84.71	−.6
13.7895	15.017	268.213	48.00	36.22	84.22	83.83	−.5
3.4474	14.004	267.065	48.30	50.15	98.44	97.38	−1.1
6.8948	13.985	274.251	48.54	46.23	94.77	93.93	9
10.3421	13.971	281.423	49.06	43.00	92.06	91.31	−.8
13.7895	13.958	288.629	49.74	40.33	90.07	89.67	−.4
4.9160	12.944	283.347	49.88	60.62	110.51	106.99	−3.3
6.8948	12.933	288.518	50.22	56.10	106.31	104.85	−1.4
8.6184	12.925	293.012	50.44	52.78	103.21	102.07	−1.1
10.3421	12.918	297.507	50.83	49.93	100.76	100.07	−.7
13.7895	12.905	306.513	51.49	45.28	96.77	95.46	−1.4
4.9160	11.973	292.548	51.49	80.27	131.76	129.01	−2.1
6.8948	11.961	298.929	51.96	70.21	122.17	*109.17	*−11.9
8.6184	11.954	304.461	52.15	63.61	115.76	113.57	−1.9
10.3421	11.946	309.999	52.47	58.39	110.86	109.53	−1.2
13.7895	11.933	321.071	53.35	50.62	103.97	103.25	−.7
5.6468	5.883	314.096	62.30	404.02	466.31	501.97	7.1
6.8948	9.166	316.310	57.35	171.78	229.12	*197.27	*−16.1
8.6184	9.158	326.292	56.89	116.33	173.22	164.57	−5.3
4.6678	3.274	310.787	55.29	94.73	150.03	149.97	−0
4.9160	3.272	316.685	54.35	80.07	134.42	134.19	−.2
5.1711	3.271	322.812	53.89	69.26	123.15	123.24	.1
4.9160	11.047	298.730	53.57	116.03	169.59	162.31	−4.5
6.8948	11.035	306.527	53.59	91.41	145.00	142.31	−1.9
8.6184	11.027	313.282	53.56	78.02	131.58	127.85	−2.9
10.3421	11.020	320.026	53.94	68.58	122.53	119.45	−2.6
12.0658	11.013	326.749	54.30	61.55	115.85	115.11	−.6
5.6468	7.888	311.185	63.62	509.77	573.39	527.29	−8.7
6.8948	7.880	320.609	60.60	217.16	277.77	273.85	−1.4
6.8948	20.286	131.554	45.81	24.64	70.45	69.02	−2.1
13.7895	20.261	135.496	45.14	24.33	69.47	69.04	−.6

* Some of Furtado’s values [18] may be in error by as much as 10 percent, see text.

## Footnotes

1 Figures in brackets indicate the literature references at the end of this paper.
